# Polyethylene Glycol as Additive to Achieve N-Conductive Melt-Mixed Polymer/Carbon Nanotube Composites for Thermoelectric Application

**DOI:** 10.3390/nano12213812

**Published:** 2022-10-28

**Authors:** Beate Krause, Petra Pötschke

**Affiliations:** Leibniz-Institut für Polymerforschung Dresden e.V. (IPF), Hohe Str. 6, 01069 Dresden, Germany

**Keywords:** thermoelectric, polymer composites, nanotubes, electron doping

## Abstract

The development of thermoelectric (TE) materials based on thermoplastic polymers and carbon nanotubes is a focus of current TE research activities. For a TE module, both p- and n-conductive composites are required, whereby the production of n-conductive materials is a particular challenge. The present study investigates whether adding polyethylene glycol (PEG) as n-dopant during the melt-mixing of the conductive composites based on polycarbonate, poly(ether ether ketone), or poly(butylene terephthalate) with singlewalled carbon nanotubes (0.5 to 2 wt%) is a possible solution. It was shown that for all three polymer types, a change in the sign of the Seebeck coefficient from positive to negative could be achieved when at least 1.5 wt% PEG was added. The most negative Seebeck coefficients were determined to be −30.1 µV/K (PC), −44.1 µV/K (PEEK), and −14.5 µV/K (PBT). The maximal power factors ranged between 0.0078 µW/m·K^2^ (PC), 0.035 µW/m·K^2^ (PEEK), and 0.0051 µW/m·K^2^ (PBT).

## 1. Introduction

Currently, more and more attention is being paid to environmentally friendly and sustainable energy production. One way to achieve this is the application of thermoelectrics (TEs) using the Seebeck effect. In this approach, a thermoelectric voltage is generated from a temperature gradient acting on a conductive material, which can serve as an energy source for various low-energy applications, such as wireless sensors for the Internet of Things [1,2,3]. In order to characterize the thermoelectric properties, the Seebeck coefficient (S) is calculated, which represents the ratio between the thermoelectric voltage (U) generated and the applied temperature difference (dT). With the S-value and the electrical conductivity (σ) of the material, the power factor (PF) can be calculated using the following equation: PF = S^2^·σ [2]. A positive Seebeck coefficient indicates a p-type (electron-withdrawing) and a negative Seebeck coefficient an n-type (electron-donating) material. In order to achieve high TE performance, a high Seebeck coefficient S and high electrical conductivity σ are desirable. However, these parameters are heavily interrelated.

One focus of development is now to replace currently mainly used expensive metal alloys, such as bismuth telluride and similar compounds [4,5], by electrically conductive polymer composites (CPCs). Such CPCs are based on insulating thermoplastic polymer matrices filled with electrically conductive fillers such as carbon-based materials like carbon nanotubes (CNTs), graphite (G), expanded graphite (EG), or graphene nanoplatelets (GNPs). In the production process of the composites, melt-mixing is especially favorable, as it is an equally well scalable and environmentally friendly process compared to solution mixing. Intrinsically, electrically conducting polymers such as PEDOT, polyaniline, pure carbon materials, or their mixtures [6,7] are also used for TE applications, but are not considered in this study.

Seen as p- and n-type materials are needed for effective TE generators (TEGs), a focus is to generate both conduction types, if possible with the same material. For the example of singlewalled carbon nanotubes (SWCNTs), in the literature a wide variety of additives are being investigated, which enable the change of conduction type from p-type to n-type [8,9,10]. Nonoguchi et al. [8] focused on organic compounds that contain benzene rings and phosphorus or, e.g., imidazole, which partly led to negative Seebeck coefficients. Piao et al. [9] brought SWCNTs into contact with solutions of polymers such as poly(methyl methacrylate) (PMMA), polystyrene (PS), polycarbonate (PC), poly(styrene sulfonic acid) (PSSA), poly(vinyl alcohol) (PVA), poly(ethyleneimine) (PEI), poly(vinylidenefluoride) (PVDF), and poly(vinylpyrrolidone) (PVP). They reported that the positive Seebeck coefficient of such treated SWCNTs remains unchanged by the addition of PC or PMMA, whereas PVA, PS, and PVDF led to slightly higher Seebeck coefficients. Only the addition of PVP and PEI resulted in a negative S-value as a result of doping of the SWCNTs by the polymer. Some of the polymers investigated by Piao et al. [9] are typical thermoplastic polymers that can also be processed into conductive composites by means of melt-mixing by the introduction of CNTs. PC, PMMA, PS, and PVDF are particularly noteworthy. In melt-mixed composites, similar trends to those observed by Piao et al. [9] could be observed with regard to the change in the sign of the S-value after mixing the CNTs with the polymers [11].

Xiao-Gang et al. [10] reported that the treatment of CNT fibers with a PEI solution changed the S-value from 76 µV/K to −72.4 µV/K. Ito et al. [12] treated SWCNTs with polyethylene glycol (PEG) and with the ionic liquid 1-butyl-3-methylimidazolium hexafluorophosphate ([BMIM]PF_6_) during a solution mixing process. While PEG addition slightly reduced the Seebeck coefficient of the SWCNTs (48 µV/K up to ca. 44 µV/K at 0.1 wt% PEG), a negative S-value of −49.1 µV/K was achieved with the addition of [BMIM]PF_6_.

For composites based on thermoplastic polymers prepared via solution mixing, PEI is also described as an additive that can change the S-value from positive to negative. Hewitt et al. [13] reported on PVDF/20 wt% SWCNT composite films whose S-value of 50 µV/K changed to −46 µV/K after adding 10 wt% PEI. Additionally, for fibers consisting of SWCNT:PVA (ratio 1:2), the addition of PEI led to a change in the Seebeck coefficient from 39.5 µV/K to −48 µV/K at 30 wt% PEI related to SWCNTs [14]. 

These approaches to change the conduction type of CNTs by adding low molecular additives are now to be transferred into melt-mixed composites. A major hurdle is that the additives must survive the melt processing at high temperatures without significant molecular degradation or even decomposition. Depending on the polymer type, during melt-mixing, temperatures of up to 360 °C are reached for short periods. This severely limits the choice of additives that have already been investigated during solution mixing. 

For imidazolium-based ionic liquids, this approach to change the Seebeck coefficient sign was successfully implemented in melt-mixed polypropylene (PP)/SWCNT composites [15]. Depending on the side groups of imidazolium ring and the anion, the Seebeck coefficient changed from 49.3 µV/K (PP/2 wt% SWCNT Tuball™) to S-vales between −17.8 and −27.6 µV/K for composites with ionic liquids (ILs) [15]. However, no switching was found when the IL 1-methyl-3-octylimidazolium tetrafluoroborate was added in PP/2 wt% Tuball composites [15] and PP composites with 2 wt% containing other kinds of SWCNTs than Tuball™ [16]. 

Another additive applied for the purpose of changing the conduction type of the composites is PEG. It is water soluble, non-toxic, and is used as an adjuvant in many cosmetics, foods, and medicines. The addition of PEG was already studied in melt-mixed PP/SWCNT composites [17,18]. The positive Seebeck coefficient of PP/0.8 wt% SWCNT + 5 wt% copper oxide at 45 µV/K could be changed to the negative value of −44.6 µV/K when 4 wt% PEG was added [17]. Simultaneously, with the addition of PEG, the volume conductivity increased from 0.003 S/cm (without PEG) to 0.02 S/cm (4 wt% PEG). Furthermore, Luo et al. [19] described the switching of the sign of the S-value after the addition of 4 wt% polyoxyethylene-20-cetyl ether into a PP/2 wt% SWCNT composite, resulting in an S-value of −38 µV/K. In addition, PEG is quite commonly used as a surfactant in the preparation of nanotube dispersions [20,21,22] or as a compatibilizer in melt-mixed composites [23,24] with the aim of improving filler dispersion. Zou et al. [24] successfully used a low molecular weight PEG in the melt of poly(L-lactide) to improve the dispersion as well as lower the rheological percolation threshold of functionalized multiwalled CNTs (MWCNTs). Müller et al. [23] added PEG of different molar masses to linear low density polyethylene/MWCNT composites and achieved a reduction in the remaining agglomerate number and a significant decrease in both the electrical percolation threshold and the volume resistivity values. The examination of the CNT nanodispersion by means of transmission electron microscopy showed that the MWCNTs without PEG are present as both individual CNTs and agglomerates. After PEG addition, however, a homogeneous CNT dispersion without remaining agglomerates is found. The improvement of CNT dispersion and decrease in volume resistivity were more pronounced when PEG with a lower molar mass was added.

In this study, PEG was used as an additive in melt-mixed polymer composites filled with SWCNTs. The aim was to change the Seebeck coefficient from positive to negative values in order to obtain suitable n-type materials for the later use in TEGs, as it was shown before for the polymer matrix of PP [17]. To explore the generality of this approach, three other polymer types that are both amorphous and partially crystalline with different polarities were used, namely polycarbonate, poly(butylene terephthalate), and poly(ether ether ketone). The miscibility of these polymers and PEG was studied by means of morphological observations.

## 2. Materials and Methods

### 2.1. Materials

**Figure 1:**
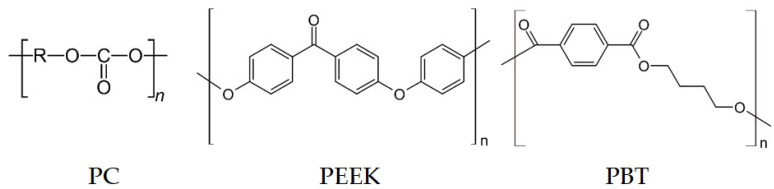
Chemical structure of polymers used in this study.

Three different kinds of commercially available polymers were selected for this study. Polycarbonate (PC) Makrolon 2600 granules (Covestro AG, Leverkusen, Germany), poly(ether ether ketone) (PEEK) Vestakeep 1000P powder (Evonik Industries AG, Essen, Germany), and poly(butylene terephthalate) (PBT) Ultradur B4500 granules (BASF SE, Ludwigshafen, Germany) were used (Figure 1).

The carbon nanotubes selected for this study are SWCNT Tuball^TM^ (OCSIAl, Luxembourg) with a carbon content of 75% [25,26]. RAMAN spectra of this CNT type are published in the supporting information of [27]. Thermoelectric parameters of the SWCNTs powder were described in [11].

Additive polyethylene glycol (PEG, Mn = 10,000 g/mol, CAS 25322-68-3) from Sigma Aldrich was used. The PEG granules are solid at 25 °C and melt at 65 °C. 

### 2.2. Methods

The composites were prepared using a conical twin-screw micro-compounder Xplore 15 (Xplore, Sittard, The Netherlands) with a volume of 15 cm^3^. The fillers were incorporated into the molten polymer and dispersed as the material was conveyed along the screws. It is important that the SWCNTs and PEG are pre-mixed by shaking in a glass. The SWCNT/PEG premix was filled into the microcompounder alternately with the polymer granulate. After cooling down, the obtained extruded strands were compression moulded into plates using the hot press PW40EH and a mask to define the outer shape of 60 mm diameter and the thickness of the plates of 0.5 mm at temperatures corresponding to the mixing temperature. The preparation conditions for melt compounding and compression molding are listed in Table 1. The processing temperatures are far above the glass transition temperatures, which are 40–60 °C (PBT), 148 °C (PC), and 150 °C (PEEK).

**Figure 2:**
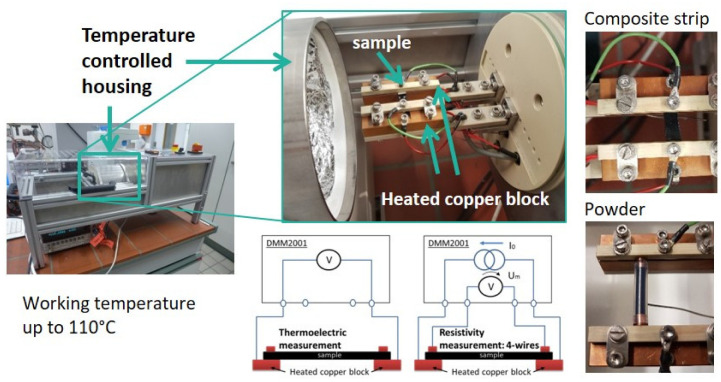
Photos and schema of the used thermoelectric measurement equipment.

The thermoelectric (TE) characterization was carried out in a measuring device developed and constructed at IPF Dresden (see Figure 2) [28]. The measuring temperature was set to 313.2 K (40.0 °C), with eight temperature variations up to ±8 K. Thus, measurements were taken at the following temperature differences : 32–40 °C, 34–40 °C, 36–40 °C, 38–40 °C, 42–40 °C, 44–40 °C, 46–40 °C, and 48–40 °C. The mean values and standard deviations of the Seebeck coefficients were calculated from measurements on two to four strips each. The individual values are composed of eight measured temperature differences, the measurement of which was repeated up to five times. The measurements of the electrical volume resistivity were conducted using the same equipment, applying the 4-wire technique on the same two samples. The mean values of resistivity were calculated from the results of two to four strips, on each of which ten measurements were performed. The measurement of thermovoltage and resistance was performed using the Keithley multimeter DMM2001 (Keithley Instruments, Cleveland, OH, USA). The samples were strips (width approx. 5 mm, thickness approx. 0.3 mm) cut from the compression molded plates and coated with conductive silver at their ends. The free sample length between the silver coated ends was 12 mm. 

For the investigation of CNT macrodispersion in the composites by transmission light microscopy (TLM), thin cuts (thickness 5 µm) were prepared at room temperature from the the extruded strands with a microtome RM2265 (Leica Mikrosysteme Vertrieb GmbH, Bensheim, Germany) equipped with a diamond knife. The cuts were fixed on glass slides using the aqueous mounting medium Aquatex^®^ (Sigma-Aldrich, Steinheim, Germany). The TLM investigations were performed with a microscope BX53M combined with a camera DP74 (Olympus Deutschland GmbH, Hamburg, Germany). 

Scanning electron microscopy (SEM) images of the samples were acquired using an ULTRA Plus (Carl Zeiss AG, Oberkochen, Germany) scanning electron microscope at 3 kV acceleration voltage using the SE2 detector. Cryo-fractured surfaces of strands and plates were observed. All samples were sputtered with a 3 nm platin film.

Thermogravimetric analysis (TGA) was performed on PEG granules by using a Q 5000 analyzer (TA Instruments, Hüllhorst, Germany) in air and nitrogen atmosphere. The heating rate was 10 K/min, and a temperature range from 25 °C up to 800 °C was applied. The test in nitrogen was intended to simulate the additive being encapsulated shortly after it was added to the polymer melt, thus practically excluding contact with the air.

## 3. Results

### 3.1. Thermal Degradation of Polyethylene Glycol

To begin, the thermal stability of the PEG was tested by means of TGA, especially at the processing temperatures of PC, PBT, and PEEK. 

**Figure 3:**
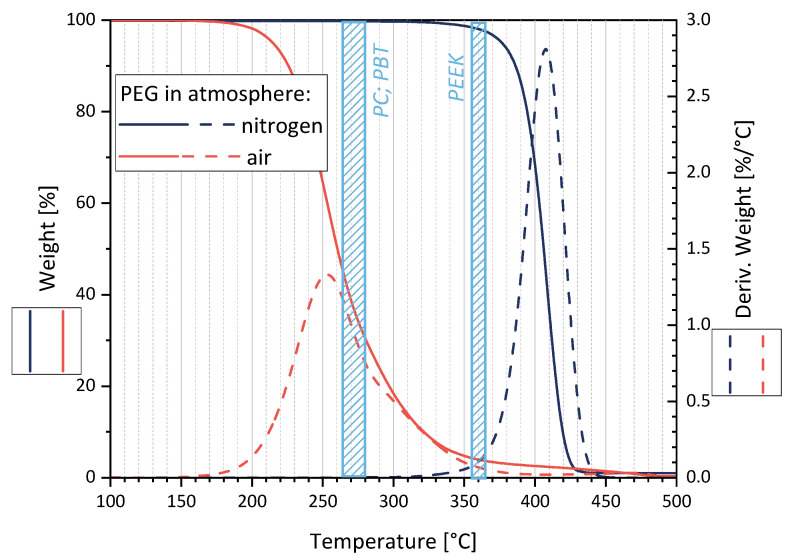
Thermal degradation behavior of PEG as studied by TGA in air and nitrogen atmosphere. The processing temperature ranges for the matrix polymers PC, PBT, and PEEK are marked as blue areas.

Figure 3 shows a maximal degradation at 255 °C (170–380 °C) in air and 410 °C (340–450 °C) in nitrogen. Assuming that PEG is encapsulated in the polymer melt (PC, PBT, or PEEK) shortly after feeding into the microcompounder, thereby avoiding contact with the air, the TGA measurements in N_2_ suggest that no significant molecular PEG degradation takes place during melt-mixing.

### 3.2. Polymer Composites with SWCNTs

**Figure 4:**
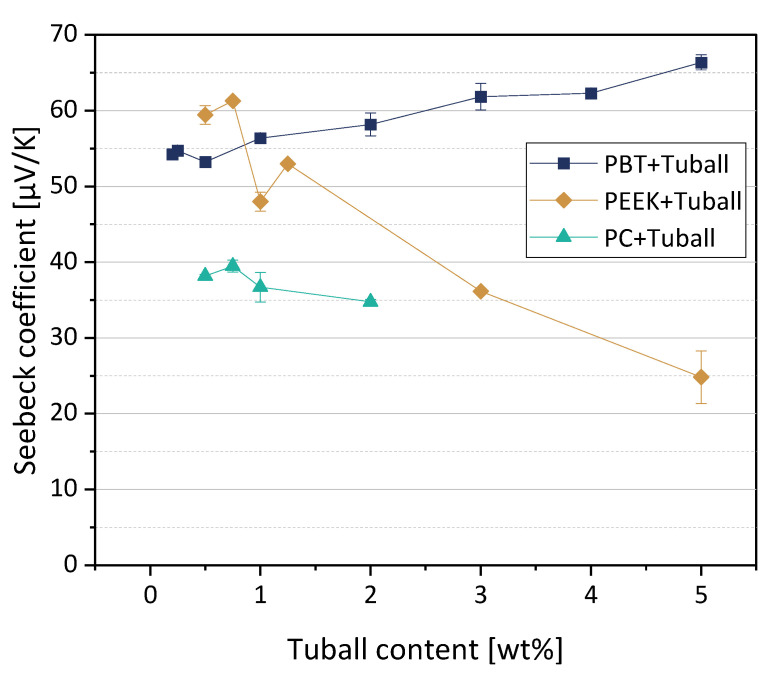
Seebeck coefficients (at 40 °C) of PC, PEEK, and PBT-based composites filled with SWCNT Tuball^™^ (values based on Refs. [11,27], newly compiled. Adapted with permission from reference [27]. Copyright 2022 American Chemical Society).

The thermoelectric properties of melt-mixed composites based on PC, PEEK, and PBT filled with SWCNT Tuball^TM^ were reported before in [11,27]. The Seebeck coefficients of the three polymer composite series are shown in Figure 4. As already noted in [11], the polymer matrix strongly influences the value of the Seebeck coefficient if the same CNT type is incorporated. For PBT composites and also for the PEEK composites with low CNT contents, the Seebeck coefficients measured are significantly higher than the S-value of the SWCNT Tuball™ powder of 39.6 µV/K [11]. The S-values of the PC composites are in the range of the SWCNTs powder.

On the other hand, the CNT type itself also influences the Seebeck coefficient value of the composite. The incorporation of MWCNTs, such as the types Nanocyl NC7000 or branched CNSPEG, leads to significantly lower S-values in the composites compared to SWCNT Tuball™, which corresponds with the order of the S-values of CNT powders. In [11], the S-values of the MWCNT powders were determined to be 6.3 µV/K for MWCNTs of the type NC7000 and 10.1 µV/K for MWCNTs of the type CNSPEG. For PC composites, only about 9 µV/K (1–2 wt% NC7000) or about 16 µV/K (0.5–2 wt% CNSPEG) could be achieved [27]. In the case of PEEK composites, only about 7 µV/K (3–5 wt% NC7000) or about 7–16 µV/K (0.5–5 wt% CNSPEG) could be measured [27]. Even in the case of PBT, the S-values of the composites were only 6.8 µV/K (2 wt% NC7000) or 15.7 µV/K (2 wt% CNSPEG) when MWCNTs were incorporated [11]. 

SWCNTs of the type Tuball™ are therefore much more relevant as conductive filler for the production of composites with p-type behavior than the investigated MWCNTs, because significantly higher Seebeck coefficients can be achieved.

### 3.3. Effects of Polyethylene Glycol as Additive in Polymer Composites with SWCNTs 

#### 3.3.1. Polycarbonate-Based Composites

**Figure 5:**
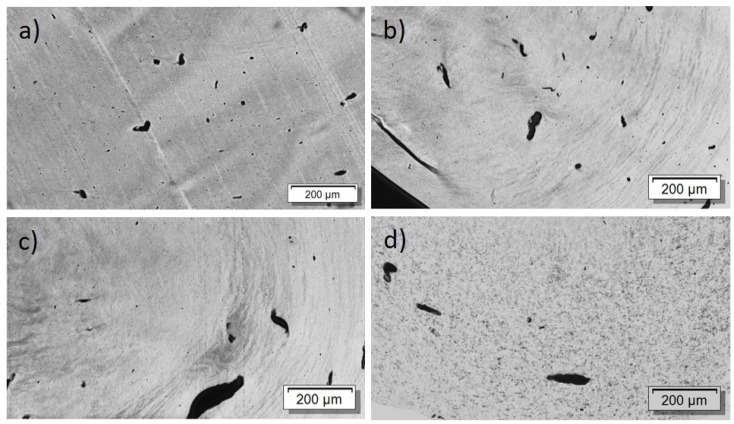
Transmission light microscopy images of PC/2 wt% Tuball^TM^ with different amounts of PEG: (**a**) without PEG, 2 wt% PEG (**b**), 3 wt% PEG (**c**), 8 wt% PEG (**d**).

**Figure 6:**
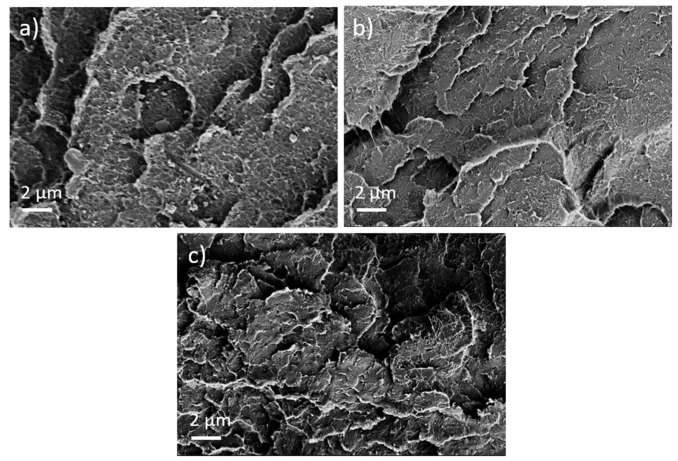
Scanning electron microscopy images of cryo-fractured surfaces of (**a**) PC/2 wt% Tuball^TM^ without PEG, (**b**) with 3 wt% PEG, and (**c**) with 8 wt% PEG.

First, the effect of PEG addition on CNT dispersion and distribution in a macro and nanoscale was studied. The TLM images of extruded strands (Figure 5) show that, at all PEG concentrations, remaining strands such as SWCNT agglomerates, similarly to those observed in previous investigations in PP composites [17], are visible. Thus, at the macroscale, no dispersion effect of the PEG is seen. By increasing PEG content, however, cloud-like light and dark grey areas are made evident, which may be a hint of the immiscibility of PEG and PC. SEM studies were carried out to further study the miscibility of PC and PEG. Figure 6 shows a PC/2 wt% SWCNT composite without and with 3 and 8 wt% PEG. The fracture surface looks identical for both samples and, especially, no additional spherical particles originating from dispersed PEG can be seen. From SEM analysis, it can be concluded that PC and PEG are miscible at the solid state.

The thermoelectric parameters of the PC/Tuball composites with PEG are summarized in Table 2. While the PC composites with 0.75 wt%, 1 wt%, and 2 wt% SWCNTs show positive S-values in the range between 36.5 and 39.5 µV/K, the addition of PEG leads to negative Seebeck coefficients for all SWCNT concentrations. However, the addition of the low amount of 1.5–2 wt% PEG resulted in S-values of only −13 to −18 µV/K. With a further increase in the PEG content to 3–8 wt%, much higher negative S-values of −27 to −30 µV/K were measured, whereby the S-value showed no clear correlation to the PEG content. Saturation seems to have been reached at 5 wt% PEG addition (at 2 wt% SWCNTs), resulting in a maximal negative S-value of −30 µV/K. This sample also shows the highest PF-value among all the PEG-modified composites of 7.6 × 10^−3^ µW/(m·K^2^). Interestingly, the electrical conductivity of the PC/Tuball composites increased (in most cases) with PEG addition, especially seen at 2 wt% SWCNTs. This effect could be related to an improved SWCNTs nanodispersion due to the low viscosity of molten PEG, which can enhance the wetting process of the SWCNTs by the polymer matrix during the melt-mixing process.

#### 3.3.2. PEEK-Based Composites

**Figure 7:**
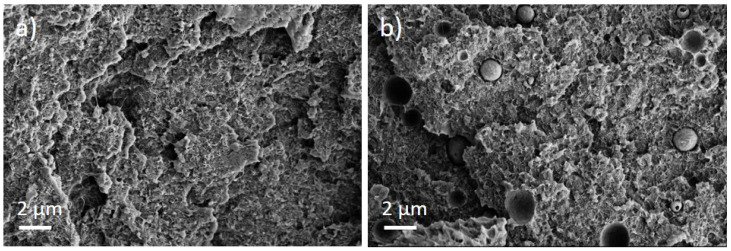
Scanning electron microscopy images of cryo-fractured surface of (**a**) PEEK/1 wt% Tuball™ without PEG and (**b**) with 2 wt% PEG.

**Figure 8:**
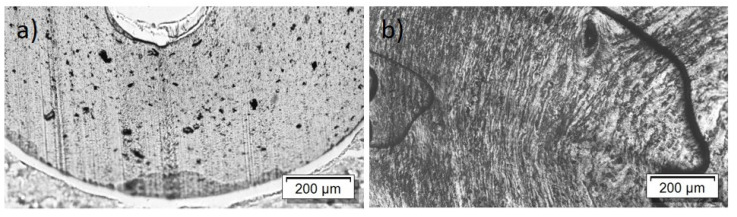
Transmission light microscopy images of PEEK/1 wt% Tuball^TM^ with different amounts of PEG: (**a**) without PEG, 2 wt% PEG (**b**).

The PEEK/Tuball composites were studied at 0.5, 0.75, and 1 wt% SWCNTs addition and 1–3 wt% PEG was applied. The SEM image of a cryo-fractured surface (Figure 7) shows, in contrast to the PC composites with PEG, spherical particles and round depths of about 2 µm diameter when PEG was added into the composite. Such particles are assigned to PEG and indicate that PEG and PEEK are not miscible at room temperature. The light microscopic study exhibits, similar to PC and PBT composites, large, stretched remaining SWCNT agglomerates in the composites (Figure 8). If PEG was added, then streaks of light grey (little or no CNTs) and dark grey (with more CNTs) are clearly visible, confirming immiscibility.

The thermoelectric results are summarized in Table 3. The results show that, in PEEK composites as well, a switching from p-type to n-type behavior was achieved with PEG addition. Similar to PC/Tuball composites, more than 1 wt% PEG should be added to generate a negative S-value of the composites with 0.5 to 1 wt% SWCNTs. The PEG addition again leads (in most cases) to an increase in the electrical volume conductivity of the composites. The highest negative Seebeck coefficient achieved was −44.1 µV/K for PEEK/0.75 wt% Tuball + 2 wt% PEG, which also resulted in the highest PF among all PEG-modified composites of 2.7 × 10^−2^ µW/(m·K^2^). When looking at the mean values of electrical conductivity and Seebeck coefficient, the relatively high standard deviations are remarkable. As described in the experimental part, measurements were taken on two to four different specimens. Even if cut from the same pressed plate at different positions, the values of the different strips differed, which is assumed to be caused by some inhomogeneity within the blend structure of the samples.

It can be concluded that the fact that PEEK and PEG are immiscible in the solid state does not seem to play a role in the doping effect of PEG on the CNTs. For the PP/Tuball + PEG composites described before in the literature [17], the immiscibility of PP and PEG was also concluded from the droplet-like structures of PEG in PP; nevertheless, negative S-values were measured after the addition of PEG.

#### 3.3.3. PBT-Based Composites

Analogous to the investigations on PC and PEEK composites with Tuball™, it was also investigated, for PBT-based composites, whether PEG incorporation can lead to a negative Seebeck coefficient. 

**Figure 9:**
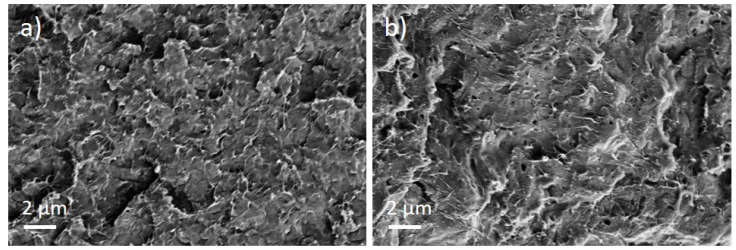
Scanning electron microscopy images of cryo-fracture of (**a**) PBT/2 wt% Tuball™ without PEG and (**b**) with 3 wt% PEG.

**Figure 10:**
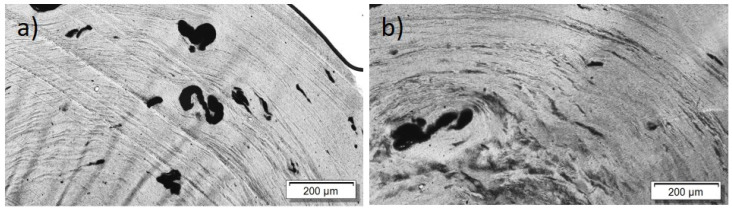
Transmission light microscopy images of PBT/2 wt% Tuball™ with different amounts of PEG: (**a**) without PEG and (**b**) 2 wt% PEG.

For the PBT/Tuball composites without and with PEG, the SEM images show a homogeneous CNT nanodispersion (Figure 9). Small hollows can be seen when PEG is present in the composite. This indicates immiscibility between PBT and PEG. The transmission light microscopy images show again the typical large remaining agglomerates independent of the PEG addition (Figure 10), indicating a relatively worse macrodispersion.

The Seebeck coefficients shown in Table 4 prove that the approach of adding PEG to change the sign of the Seebeck coefficient also works for PBT-based composites. However, the S-values of about −14 µV/K are relatively low. Since no further increase in the negative S-value was observed for the PC/Tuball composites when increasing the PEG content from 3 to 5 wt%, this material development was not pursued further. Again, the electrical volume conductivity is increased when adding PEG. The maximal PF-value achieved with n-type composites is 2.6 × 10^−3^ µW/(m·K^2^) for PBT/2 wt% Tuball + 5 wt% PEG.

## 4. Discussion

When the SWCNTs of the type Tuball™ were incorporated into different thermoplastic matrices (PC, PEEK, and PBT) with up to 5 wt%, positive Seebeck coefficients were generally measured. The magnitude of the S-values of the composites was different depending on the matrix polymer. In particular, the composites based on PBT and PEEK showed significantly higher S-values compared to the pure SWCNTs powder (39.6 µV/K [11]). In contrast, the Seebeck coefficients of the PC-based composites are approximately in the range of the S-value of the pure SWCNTs. Additionally, for PP/SWCNT composites reported before by Luo et al. [29], the S-value of 35.6 µV/K for a composite with 2 wt% SWCNTs is near to that of the SWCNTs powder. The S-value of these composites decreased with increasing SWCNTs content up to 26.0 µV/K (at 6 wt% SWCNTs). 

When considering the chemical structures of the polymers, it is noticeable that both PBT and PEEK, the polymers which resulted in their composites in enhanced Seebeck values, have an extended π-electron system due to the benzene rings, ether, and carboxyl groups they contain (Figure 1). This has the consequence of electrons being drawn from the nanotubes into the polymer and thus the p-type character of the conductive network is enhanced. This may explain increasing Seebeck coefficients in those composites compared to the pure SWCNT material. For PEEK/carbon nanofiber composites, Paleo et al. [30] modelled such an effect. Using a quantum chemical computer model, an electron-withdrawing effect was inferred from the PEEK molecules in contact with the outermost graphene layers of the carbon nanofibers. Due to the similar structure of nanotubes to graphene, an electron-withdrawing effect of the PEEK is also assumed in the study discussed here, which results in the increase in the Seebeck coefficient of the composites compared to the CNT powder. 

The structural formulas of PC or PP do not contain groups that can strongly attract electrons. This agrees with the quite similar Seebeck coefficients of the composites and the SWCNTs powder. Piao et al. [9] also described no significant doping effect when SWCNTs were treated by PC solutions. The assumptions on the influence of the polymer matrix on the conducting type and intensity of the nanotubes are to be substantiated by calculations in a further study.

The addition of PEG as an additive during the melt-mixing of SWCNT–polymer composites led to a change in the Seebeck coefficient in the polymers PC, PEEK, and PBT from positive values to negative ones. This finding is in agreement with the results already described for PP/SWCNT composites [17]. The result proves that PEG is also suitable for acting as an n-dopant in melt-mixed composites for different polymer matrices. 

In order to perform such doping, the process of incorporating the PEG additive is of great importance in the production of the composites. In the Experimental Section, it was described that the CNTs and the PEG were first premixed and then added to the molten polymer. Interestingly, in the trials, to first premix the polymer and the CNTs and add the PEG afterwards, or if all three components were filled in the microcompounder alternately at the beginning of the mixing, the resultant composites showed a positive Seebeck coefficient. Therefrom, it can be concluded that the wetting of the nanotubes with PEG is of decisive importance for the conduction type of the composite. During the premixing, an evident surface PEG layer is formed around the tubes or tube bundles which either in that premixing step or later under the influence of the temperature in the mixing process dopes the CNTs. To verify this thesis, a composite of PEG with 2 wt% Tuball was prepared and a Seebeck coefficient of −2.0 +/− 0.7 µV/K at a conductivity of 2.3 S/m was determined. This result clearly underlines that PEG can be effective as an n-dopant.

Furthermore, hardly any dependencies can be identified with regard to the PEG content or the ratio between SWCNTs and PEG. For both the PC and PEEK composites, a SWCNT:PEG ratio of 1:2 was set by varying the SWCNTs content. For both polymers, the S-value becomes more and more negative with increasing SWCNTs content. In detail, the PEEK/0.5 wt% Tuball + 1 wt% PEG has a significantly reduced S-value, which is still positive at 13.9 µV/K. At the same SWCNT:PEG ratio and higher SWCNTs contents of 0.75 and 1.0 wt%, the Seebeck coefficients of the PEEK composites are negative at −22.4 µV/K and −35.0 µV/K, respectively (Table 3). For the PEEK composites with higher SWCNT:PEG ratios, negative S-values were always determined, with the values being similar and the large standard deviations indicating inhomogeneities in the composites. For the PC composites, the trends are comparable. Especially for PC/2 wt% Tuball, it can be seen that a higher PEG content leads to a plateau with values between −26.4 and −30.1 µV/K (Table 2). For the PBT/2 wt% Tuball composites, the addition of 3 or 5 wt% PEG led to the same Seebeck coefficient of −14 µV/K, which also suggests that a plateau is developed (Table 4). It can be concluded that for the sufficient wetting of the SWCNTs during melt-mixing, a certain amount of PEG must be present. However, excess PEG does not further improve the negative Seebeck coefficient of the composite. This could be an indication that the CNT surface is then saturated with PEG molecules. The absolute PEG content in the different composites does not seem to have any influence on the S-value. For example, depending on the SWCNT content, 2 wt% PEG addition leads to S-values of −22.6 µV/K, −44.1 µV/K and −35.0 µV/K in PEEK, and to −18.3 µV/K and −13.0 µV/K in PC.

Furthermore, it was found that the addition of PEG generally slightly enhances the electrical conductivity in all three polymer composites. This indicates that the PEG-wetted SWCNTs are apparently better dispersed and more easily distributed in the molten polymer matrix and that the conductive network is therefore better developed. PEG thus has a second function as a dispersing agent which is in good agreement with the previous findings in the literature [23]. 

In addition to the dispersion and distribution of the CNTs, the miscibility of the applied polymers and PEG was also investigated by morphological observations. The light microscopic images of the composites show many large elongated remaining SWCNT agglomerates (Figure 5 and Figure 10). This poor dispersibility of the SWCNT Tuball™ material has been reported before and was found to also be typical in PP [15,31], acrylonitrile butadiene styrene (ABS) [32], and polyamide 6 [32,33]-based composites. From the SEM study, it was concluded that PC and PEG are miscible in the solid state, in which the measurements were performed, but PBT and PEEK are not. In PBT and PEEK-based composites with PEG, small hollows or dispersed particles assigned to PEG were observed on the cryofractured surfaces, which was not observed in PC-based composites. Therefrom, it can be concluded that the miscibility of PEG with the matrix polymers does not have an influence on the effect of PEG on the TE properties of the composites. This supports the assumption that the wetting of the SWCNTs at the beginning of the melt-mixing process is the most important step to obtain such sign switch in the Seebeck coefficient.

Even though the formulations are not directly comparable, the highest negative Seebeck coefficients achieved for each of the polymer matrices studied here are slightly lower than those reported in our previous paper for PP-based composites [17]. For the PP composite with 2 wt% Tuball, 5 wt% cupper oxide, and 10 wt% PEG, values of the Seebeck coefficient of −56.6 μV/K and power factor of 0.078 µW/(m·K^2^) were found, whereas in our new study S-values of −30.1 µV/K (PC), −44.1 µV/K (PEEK), and −14.5 µV/K (PBT) were determined. Maximal power factors were also lower, ranging across 0.0078 µW/m·K^2^ (PC), 0.035 µW/m·K^2^ (PEEK), and 0.0051 µW/m·K^2^ (PBT). The reasons for those differences will be studied in further investigations.

In addition, n-type composites could also be reached without the use of PEG, for example when using nitrogen doped n-type multiwalled CNTs (N-MWCNTs) [34]. With such an approach, in melt-mixed PP-based composites, a maximum negative Seebeck coefficient of −22.9 µV/K and a maximum power factor of 0.0011 µW/(m·K^2^) was determined (PP/5 wt% N-MWCNTs). This shows that negative Seebeck values of N-MCNTS can be transferred into composites. Furthermore, certain polymer matrices containing nitrogen-containing groups, such as different kinds of polyamides (PAs) and acrylonitrile butadiene styrene (ABS), are shown to induce negative Seebeck coefficients when combined with SWCNTs of the type Tuball [11]. For such combinations, maximum negative Seebeck coefficients of −57.1 µV/K (ABS/0.5 wt% CNT) and −59.8 µV/K (PA6/1 wt% CNT) were reached. The maximum power factors were 0.14 µW/(m·K^2^) (ABS/5 wt% CNT and PA6/5 wt% CNT) [11]. This strongly underlines the importance of the doping effect of the polymer matrix on SWCNTs.

## 5. Summary

The investigations show that the use of PEG in melt-mixing of SWCNTs into different polymer types can result in a switch of the sign of the Seebeck coefficient, not only for the previously described case of PP, but also for the thermoplastic polymers PC, PEEK, and PBT. The results show that there seems to be an optimal PEG content depending on the CNT concentration in order to reach negative Seebeck coefficients. Sufficient surface wetting of the SWCNTs with PEG during the beginning of melt-mixing is crucial, which can only be ensured by premixing the CNTs with the PEG. The miscibility of the PEG with the polymer matrix seems to not be of importance for the effect of switching the conduction type of the composites. The best values achieved in this study on composites with negative Seebeck coefficients were −44.1 µV/K for PEEK/0.75 wt% Tuball + 2 wt% PEG, and the highest PF among all PEG-modified composites of 0.035 µW/(m·K^2^) for PEEK/1 wt% Tuball + 2 wt% PEG. In further studies, the electron-withdrawing or electron-donating properties of the different thermoplastic polymers and polyethylene glycol on the SWCNTs are planned to be investigated in more detail and quantitatively.

## Figures and Tables

**Table 1:** Conditions of melt compounding and compression molding using Xplore 15 microcompounder and hot press PW40EH.

Polymer	Temperature (°C)	Rotation Speed (rpm)	Mixing Time (min)	Pressing Time (min)
PBT	265	200	5	1
PC	280	250	5	1
PEEK	360	250	5	1

**Table 2:** TE properties of PC composites with SWCNT Tuball and PEG in different quantities.

Composite	El. vol. Conductivity σ (S/m)	Seebeck Coeff. S (µV/K)	Power Factor PF (µW/(m·K^2^))
PC/0.75 wt% Tuball [27]	0.9	39.5 ± 0.8	1.4 × 10^−3^
PC/0.75 wt% Tuball + 1.5 wt% PEG	0.57 ± 0.1	−13.9 ± 0.4	1.1 × 10^−4^
PC/1 wt% Tuball [27]	1.0	36.7 ± 2.0	1.3 × 10^−3^
PC/1 wt% Tuball + 2 wt% PEG	1.77 ± 0.3	−18.3 ± 2.7	6.0 × 10^−4^
PC/2 wt% Tuball [27]	1.0	37.8 ± 0.3	1.2 × 10^−3^
PC/2 wt% Tuball + 2 wt% PEG	1.32 ± 0.3	−13.0 ± 0.1	2.2 × 10^−4^
PC/2 wt% Tuball + 3 wt% PEG	7.18 ± 0.4	−28.9 ± 1.0	6.0 × 10^−3^
PC/2 wt% Tuball + 4 wt% PEG	4.65 ± 0.3	−26.4 ± 1.9	3.4 × 10^−3^
PC/2 wt% Tuball + 5 wt% PEG	8.58 ± 1.4	−30.1 ± 0.7	7.8 × 10^−3^
PC/2 wt% Tuball + 8 wt% PEG	9.33 ± 1.9	−27.2 ± 0.8	6.9 × 10^−3^

**Table 3:** TE properties of PEEK composites with SWCNT Tuball and PEG in different quantities.

Composite	El. Vol. Conductivity σ (S/m)	Seebeck Coeff. S (µV/K)	Power Factor PF (µW/(m·K^2^))
PEEK/0.5 wt% Tuball [27]	2.2	59.4 ± 1.2	7.6 × 10^−2^
PEEK/0.5 wt% Tuball + 1 wt% PEG	2.70 ± 0.1	13.9 ± 0.5	5.2 × 10^−4^
PEEK/0.5 wt% Tuball + 2 wt% PEG	1.28 ± 0.5	−22.6 ± 0.6	6.6 × 10^−4^
PEEK/0.5 wt% Tuball + 3 wt% PEG	12.27 ± 2.0	−37.3 ± 0.3	1.7 × 10^−2^
PEEK/0.75 wt% Tuball [27]	1.8	61.3 ± 0.2	1.2 × 10^−2^
PEEK/0.75 wt% Tuball + 1.5 wt% PEG	4.88 ± 0.0	−22.4 ± 1:6	2.5 × 10^−3^
PEEK/0.75 wt% Tuball + 2 wt% PEG	4.73 ± 0.2	−44.1 ± 3.8	9.2 × 10^−3^
PEEK/0.75 wt% Tuball + 3 wt% PEG	0.45 ± 0.2	−36.7 ± 1.0	6.1 × 10^−4^
PEEK/1 wt% Tuball [27]	6.2	48.0 ± 1.3	7.2 × 10^−3^
PEEK/1 wt% Tuball + 1 wt% PEG	14.32± 3.2	−24.0 ± 4.2	8.3 × 10^−3^
PEEK/1 wt% Tuball + 2 wt% PEG	28.44 ± 19.9	−35.0 ± 5.1	3.5 × 10^−2^
PEEK/1 wt% Tuball + 3 wt% PEG	15.91 ± 3.9	−33.7 ± 2.1	1.8 × 10^−2^

**Table 4:** TE properties of PBT composites with SWCNT Tuball™ and PEG in different quantities.

Composite	El. Vol. Conductivity σ (S/m)	Seebeck Coeff. S (µV/K)	Power Factor PF (µW/(m·K^2^))
PBT/2 wt% Tuball [11]	2.2	59.4 ± 1.2	1.0 × 10^−2^
PBT/2 wt% Tuball + 3 wt% PEG	12.42 ± 4.2	−14.0 ± 1.1	2.4 × 10^−3^
PBT/2 wt% Tuball + 5 wt% PEG	24.01 ± 4.8	−14.5 ± 0.1	5.1 × 10^−3^

## Data Availability

The data presented in this study are available upon request from the corresponding author.

## References

[1] Poehler T.O., Katz H.E. (2016). Innovative thermoelectric materials. Innovative Thermoelectric Materials.

[2] Rowe D.M. (1995). CRC Handbook of Thermoelectrics.

[3] Yazawa K., Bahk J.-H., Shakouri A.T (2021). Thermoelectric Energy Conversion Devices and Systems.

[4] Goldsmid H.J. (2010). Introduction to Thermoelectricity.

[5] Hongmei W., Jun Y., Zhou D. Review of recent developments in thermoelectric materials. Proceedings of the 2016 International Conference on Robots & Intelligent System (ICRIS).

[6] Gayner C., Kar K.K. (2016). Recent advances in thermoelectric materials. Prog. Mater. Sci..

[7] Zhang L., Shi X.-L., Yang Y.-L., Chen Z.-G. (2021). Flexible thermoelectric materials and devices: From materials to applications. Mater. Today.

[8] Nonoguchi Y., Ohashi K., Kanazawa R., Ashiba K., Hata K., Nakagawa T., Adachi C., Tanase T., Kawai T. (2013). Systematic conversion of single walled carbon nanotubes into n-type thermoelectric materials by molecular dopants. Sci. Rep..

[9] Piao M., Alam M.R., Kim G., Dettlaff-Weglikowska U., Roth S. (2012). Effect of chemical treatment on the thermoelectric properties of single walled carbon nanotube networks. Phys. Status Solidi.

[10] Xia X.-G., Zhang Q., Zhou W.-B., Xiao Z.-J., Xi W., Wang Y.-C., Zhou W.-Y. (2021). Highly flexible and excellent performance continuous carbon nanotube fibrous thermoelectric modules for diversified applications. Chin. Phys. B.

[11] Krause B., Barbier C., Levente J., Klaus M., Pötschke P. (2019). Screening of different carbon nanotubes in melt-mixed polymer composites with different polymer matrices for their thermoelectric properties. J. Compos. Sci..

[12] Ito M., Koizumi T., Kojima H., Saito T., Nakamura M. (2017). From materials to device design of a thermoelectric fabric for wearable energy harvesters. J. Mater. Chem. A.

[13] Hewitt C.A., Montgomery D.S., Barbalace R.L., Carlson R.D., Carroll D.L. (2014). Improved thermoelectric power output from multilayered polyethylenimine doped carbon nanotube based organic composites. J. Appl. Phys..

[14] Ding T., Chan K.H., Zhou Y., Wang X.-Q., Cheng Y., Li T., Ho G.W. (2020). Scalable thermoelectric fibers for multifunctional textile-electronics. Nat. Commun..

[15] Voigt O., Krause B., Pötschke P., Müller M.T., Wießner S. (2022). Thermoelectric performance of polypropylene/carbon nanotube/ionic liquid composites and its dependence on electron beam irradiation. J. Compos. Sci..

[16] Luo J., Krause B., Pötschke P. (2017). Polymer-carbon nanotube composites for thermoelectric applications. AIP Conf. Proc..

[17] Luo J., Cerretti G., Krause B., Zhang L., Otto T., Jenschke W., Ullrich M., Tremel W., Voit B., Pötschke P. (2017). Polypropylene-based melt mixed composites with singlewalled carbon nanotubes for thermoelectric applications: Switching from p-type to n-type by the addition of polyethylene glycol. Polymer.

[18] Pötschke P., Krause B., Luo J. (2018). Melt-mixed thermoplastic polymer/carbon nanotube composites for thermoelectric applications. TechConnect Briefs.

[19] Pötschke P., Krause B., Luo J. (2019). Melt mixed composites of polypropylene with single walled carbon nanotubes for thermoelectric applications: Switching from p- to n-type behavior by additive addition. AIP Conf. Proc..

[20] Chen S., Jiang Y., Wang Z., Zhang X., Dai L., Smet M. (2008). Light-controlled single-walled carbon nanotube dispersions in aqueous solution. Langmuir.

[21] Lee J.U., Huh J., Kim K.H., Park C., Jo W.H. (2007). Aqueous suspension of carbon nanotubes via non-covalent functionalization with oligothiophene-terminated poly(ethylene glycol). Carbon.

[22] Vaisman L., Marom G., Wagner H.D. (2006). Dispersions of surface-modified carbon nanotubes in water-soluble and water-insoluble polymers. Adv. Funct. Mater..

[23] Müller M.T., Krause B., Pötschke P. (2012). A successful approach to disperse mwcnts in polyethylene by melt mixing using polyethylene glycol as additive. Polymer.

[24] Zou S., Na B., Lv R., Pan H. (2012). The plasticizer-assisted formation of a percolating multiwalled carbon nanotube network in biodegradable poly(l-lactide). J. Appl. Polym. Sci..

[25] Krause B., Pötschke P., Ilin E., Predtechenskiy M. (2016). Melt mixed SWCNT-polypropylene composites with very low electrical percolation. Polymer.

[26] Predtechenskiy M.R., Khasin A.A., Bezrodny A.E., Bobrenok O.F., Dubov D.Y., Muradyan V.E., Saik V.O., Smirnov S.N. (2022). New perspectives in SWCNT applications: Tuball SWCNTs. Part 1. Tuball by itself—All you need to know about it. Carbon Trends.

[27] Konidakis I., Krause B., Park G.-H., Pulumati N., Reith H., Pötschke P., Stratakis E. (2022). Probing the carrier dynamics of polymer composites with single and hybrid carbon nanotube fillers for improved thermoelectric performance. ACS Appl. Energy Mater..

[28] Jenschke W., Ullrich M., Krause B., Pötschke P. (2020). Messanlage zur Untersuchung des Seebeck-Effektes in Polymermaterialien–Measuring apparatus for study of seebeck-effect in polymer materials. Tech. Mess..

[29] Luo J., Krause B., Pötschke P. (2016). Melt-mixed thermoplastic composites containing carbon nanotubes for thermoelectric applications. AIMS Mater. Sci..

[30] Paleo A.J., Krause B., Soares D., Melle-Franco M., Muñoz E., Pötschke P., Rocha A.M. (2022). Thermoelectric properties of n-type poly(ether ether ketone) carbon nanofiber melt-processed composites. Polymers.

[31] Krause B., Barbier C., Kunz K., Pötschke P. (2018). Comparative study of singlewalled, multiwalled, and branched carbon nanotubes melt mixed in different thermoplastic matrices. Polymer.

[32] Kunz K., Krause B., Kretzschmar B., Juhasz L., Kobsch O., Jenschke W., Ullrich M., Pötschke P. (2019). Direction dependent electrical conductivity of polymer/carbon filler composites. Polymers.

[33] Krause B., Liguoro A., Pötschke P. (2021). Blend structure and n-type thermoelectric performance of PA6/SAN and PA6/PMMA blends filled with singlewalled carbon nanotubes. Nanomaterials.

[34] Krause B., Konidakis I., Arjmand M., Sundararaj U., Fuge R., Liebscher M., Hampel S., Klaus M., Serpetzoglou E., Stratakis E. (2020). Nitrogen-doped carbon nanotube/polypropylene composites with negative seebeck coefficient. J. Compos. Sci..

